# Time-Dependent Outcomes of Convalescent Plasma in Early COVID-19: A Single-Center Cohort with a Host–Pathogen Perspective

**DOI:** 10.3390/pathogens15010037

**Published:** 2025-12-28

**Authors:** Katarzyna Kalinowska, Patrycja Bociąga, Benita Wiatrak

**Affiliations:** 1 Department and Clinic of Pulmonology and Lung Cancer, Wroclaw Medical University, Grabiszynska 105 St., 53-439 Wroclaw, Poland; k.kalinowska@umw.edu.pl; 2 Department of Transfusion Immunology, Lower Silesian Center of Oncology, Pulmonology and Hematology, 53-439 Wroclaw, Poland; patrycja.bociaga@dcopih.pl; 3 Department of Pharmacology, Wroclaw Medical University, Mikulicza-Radeckiego 2, 50-345 Wroclaw, Poland

**Keywords:** COVID-19 convalescent plasma, time-varying exposure, landmark analysis, competing risks, host–pathogen interaction, immunosuppression

## Abstract

Background: Evidence on COVID-19 convalescent plasma (CCP) is mixed. We examined associations between CCP administration and in-hospital outcomes among patients hospitalized during early pandemic waves in Poland. Methods: We conducted a retrospective, single-center cohort study of adults hospitalized with COVID-19 between October 2020 and January 2021. Patients receiving CCP were compared with contemporaneous controls without CCP. Primary outcomes were in-hospital mortality and discharge alive. Requirement for invasive mechanical ventilation/intubation was summarized descriptively because timing of intubation was not reliably available. Group comparisons used χ^2^/Fisher’s exact tests and *t*-test/Mann–Whitney U tests as appropriate. Associations with mortality and discharge were evaluated using logistic regression: (i) a prespecified age-adjusted model and (ii) an exploratory prognostic model including in-hospital treatments and severity markers (systemic glucocorticoids, remdesivir, oxygen therapy, and antibiotic use), interpreted prognostically rather than causally. Results: The cohort included 224 patients (CCP, *n* = 92; controls, *n* = 132); outcome status was missing for eight controls. Baseline demographics, comorbidities, and admission laboratory values were broadly comparable between groups. Crude in-hospital mortality was 25% in the CCP group (23/92) versus 42% in controls (52/124; *p* = 0.010), and discharge alive occurred in 66% versus 50%, respectively (*p* = 0.022). Invasive mechanical ventilation/intubation was required in 12.0% of CCP recipients and 4.5% of controls (*p* = 0.071). In age-adjusted models, CCP was associated with lower odds of in-hospital death. In exploratory prognostic models incorporating systemic glucocorticoids, remdesivir, oxygen therapy, and antibiotic use, CCP remained associated with lower odds of death and higher odds of discharge alive. Conclusions: In this early-wave retrospective cohort, CCP administration was associated with lower in-hospital mortality and higher discharge rates. Exploratory analyses adjusted for concomitant in-hospital therapies and severity markers should be interpreted as prognostic associations rather than evidence of causal efficacy.

## 1. Introduction

The first cases of coronavirus disease 2019 (COVID-19) were reported in Wuhan, China, in December 2019. Within weeks, the infection caused by severe acute respiratory syndrome coronavirus-2 (SARS-CoV-2) had spread globally, and on 11 March 2020, the World Health Organization (WHO) declared a pandemic [1]. The outbreak placed an unprecedented burden on health care systems, necessitating rapid evaluation of therapeutic strategies [2,3].

The clinical course of COVID-19 is highly variable, ranging from asymptomatic or mild disease through moderate illness to severe and critical cases. Mild forms are characterized by symptoms such as fever, cough, myalgia, headache, anosmia, or gastrointestinal disturbances. Severe disease presents with pneumonia and hypoxemia, while critical illness may involve acute respiratory distress syndrome, septic shock, and multi-organ failure. Older age and comorbidities, including cardiovascular disease, diabetes, obesity, chronic kidney disease, chronic lung disease, malignancy, and immunosuppression, are strongly associated with an increased risk of hospitalization and mortality [4,5,6].

Over the course of the pandemic, multiple therapeutic strategies were investigated. Antiviral agents such as remdesivir [7] and later nirmatrelvir/ritonavir [8] were introduced through drug repurposing efforts. Anti-inflammatory treatments, including glucocorticoids [9] and immunomodulators such as tocilizumab [10] and baricitinib [11], became standard of care for patients with severe or critical disease. Anticoagulation was widely adopted to reduce thromboembolic complications [12]. The progressive introduction of vaccination and the emergence of less virulent viral variants, such as Omicron, substantially modified the clinical picture of COVID-19 in subsequent years [13,14].

During early SARS-CoV-2 infection, viral replication precedes the hyper-inflammatory phase, suggesting a therapeutic window in which exogenous neutralizing antibodies may improve viral clearance. The magnitude and durability of this effect depend on both pathogen characteristics (variant susceptibility) and host factors (age-related immunity, immunosuppression, baseline inflammatory response). CCP thus represents an intervention at the interface of host–pathogen biology, where time of administration and CCP-antibody titre as key determinants of effectiveness.

CCP was one of the first immunotherapeutic approaches available in the early phase of the pandemic. The rationale for its use was based on passive transfer of neutralizing antibodies from recovered individuals to acutely ill patients. Initial observational reports suggested possible benefits, including viral clearance and improved survival, particularly when administered early in the disease course. However, subsequent randomized controlled trials yielded inconsistent results, and the overall effectiveness of CCP therapy remained controversial [15]. Importantly, no large randomized clinical trials were performed in Poland, despite relatively widespread use of CCP during the first pandemic waves.

Therefore, the aim of our study was to retrospectively evaluate the effectiveness of CCP therapy in hospitalized patients with COVID-19 in our center, and to identify independent prognostic factors for mortality and hospital discharge.

## 2. Materials and Methods

### 2.1. Patient Characteristics

This study was designed as a retrospective analysis of patients hospitalized due to COVID-19 at the Department and Clinic of Pulmonology, Wroclaw Medical University, located in Lower Silesian Center of Oncology, Pulmonology and Hematology, Wroclaw, Poland, during the early phase of the pandemic. The study period covered October 2020 to January 2021 (second wave in Poland), prior to the emergence of Delta or Omicron variants. Individual-level SARS-CoV-2 variant data were not available because sequencing was not performed routinely. However, the study period corresponds to Poland’s second pandemic wave, preceding Delta/Omicron, when pre-Alpha lineages predominated; Poland’s third wave was later mainly associated with the Alpha variant.

A total of 224 adults with confirmed SARS-CoV-2 infection hospitalized during this period were included. The diagnosis was confirmed by RT-PCR and/or a SARS-CoV-2 antigen test performed on nasopharyngeal swabs. Among the entire cohort, 92 patients (41%) received COVID-19 convalescent plasma (CCP), while 132 patients (59%) were managed without this therapy.

Baseline disease severity was characterized by the highest level of respiratory support required during hospitalization and categorized as: (i) no oxygen/ventilatory support; (ii) conventional oxygen therapy only; (iii) non-invasive ventilation or high-flow nasal oxygen therapy (HFNOT); and (iv) invasive mechanical ventilation. The distribution of these categories by treatment group is reported in Table 1.

Comorbidities including hypertension, diabetes mellitus, obesity, chronic kidney disease, chronic lung disease, ischemic heart disease, vascular disease, and malignancies were extracted from medical records and are summarized in Table 1. The presence of underlying malignancy was used as a proxy for cancer; however, detailed information on active chemotherapy or other chronic immunosuppressive regimens was not captured in a standardized way and could not be reliably reconstructed from free-text documentation (see Section 2.2). Nevertheless, we were able to capture basic information on selected major in-hospital therapies (including systemic glucocorticoids, e.g., dexamethasone), whereas comprehensive details of outpatient/chronic immunosuppressive regimens remained incomplete.

### 2.2. Clinical Data

Clinical and laboratory data were obtained from medical records. Demographic variables, including age and sex, were collected, along with comorbidities such as diabetes mellitus, hypertension, chronic kidney disease, chronic lung disease, vascular disease, and malignancies. For malignancy, both solid and haematological neoplasms were captured; however, information on active chemotherapy, systemic immunosuppressive therapy (e.g., for autoimmune disease), and other forms of chronic immunosuppression was not consistently recorded in structured fields. Where such therapies were clearly documented in free-text notes, they were reviewed, but the data were too sparse and heterogeneous to be summarized quantitatively; this limitation is acknowledged in the Discussion.

Supportive therapies administered during hospitalization were analyzed, including remdesivir, systemic glucocorticoids (e.g., dexamethasone), antibiotics, oxygen therapy, non-invasive ventilation, high-flow nasal oxygen therapy (HFNOT), and intubation with invasive mechanical ventilation. Concomitant COVID-19 treatments (e.g., remdesivir, systemic glucocorticoids) were recorded for both CCP and control groups and are summarized in Table 1.

Selected laboratory parameters at admission, such as complete blood count, D-dimer concentration, creatinine, aminotransferase activity, blood glucose, and electrolytes, were also evaluated. Baseline anti-SARS-CoV-2 antibody status at admission (patient serology) was not routinely measured in this cohort and is therefore not available for analysis or stratification by serostatus.

### 2.3. CCP Characteristics and Timing Variables

CCP unit-level variables. During the early phase of the national programme, routine quantification of SARS-CoV-2–specific antibody titres or neutralizing activity was not consistently available. When present, antibody titre/reactivity information (“Titre p/c”) was extracted from unit-level documentation; however, these data were incompletely captured and reported in non-uniform formats. Therefore, donor-unit antibody levels were not incorporated into outcome models.

Exposure timing. Time from hospital admission to the first CCP transfusion was calculated in days and used as the exposure time scale in time-to-event analyses. Date of symptom onset was not consistently recorded and therefore could not be used to define the time origin. Where available, unit-level collection and issue dates were extracted to calculate the interval from donation (collection) to transfusion.

### 2.4. Statistical Analysis

Analyses were conducted in a retrospective cohort study comparing patients hospitalized for COVID-19 who received convalescent plasma (CCP) with a control group (no CCP) recruited from admissions during the same period. Continuous variables were summarized as mean (SD) or median (IQR), depending on distribution, and categorical variables as counts and percentages. Between-group comparisons used Student’s *t*-test or the Mann–Whitney U test for continuous variables and the χ^2^ test or Fisher’s exact test for categorical variables (depending on expected cell counts).

The endpoints were: (1) in-hospital death and (2) live hospital discharge; the need for intubation and mechanical ventilation was additionally reported descriptively. When information on a patient’s final hospital outcome was missing, those observations were excluded from endpoint analyses (complete-case analysis for outcomes); therefore, crude event rates and Kaplan–Meier summaries were based on patients with complete discharge status (92/92 CCP patients and 124/132 controls).

The association between CCP administration and endpoints was assessed using logistic regression, reporting odds ratios (ORs) with 95% confidence intervals (95% CIs) and *p* values. We prespecified:

(1) a basic model (primary analysis)—a model adjusted for age and CCP exposure, representing minimal adjustment for the most important prognostic factor available completely and comparably across groups;
(2) exploratory (prognostic) models—models additionally including selected exposures/interventions recorded during hospitalization (e.g., oxygen therapy and antibiotic, systemic glucocorticoids, remdesivir) as binary markers of disease severity and intensity of care. These variables were not treated as classic baseline confounders; their inclusion was descriptive/prognostic because they could occur following clinical deterioration (risk of time-dependent confounding). Consequently, the results of the exploratory models were interpreted as prognostic associations rather than evidence of a causal treatment effect.

All tests were two-sided, and statistical significance was defined as *p* < 0.05. All statistical analyses were performed using R, version 4.5.2.

## 3. Results

### 3.1. Patient Characteristics

A total of 224 patients hospitalized for COVID-19 were included, including 92 (41%) treated with CCP and 132 (59%) in the control group. The mean age of the entire cohort was 68 years (median 70). CCP-treated patients were on average slightly younger than controls (65.6 vs. 69.4 years), with the difference being borderline (*p* = 0.062). Sex distribution was similar in both groups.

The prevalence of comorbidities was comparable between groups (including hypertension, diabetes, obesity, chronic kidney disease, and ischemic heart disease), with no statistically significant differences (Table 1). Some patients had more than one comorbidity. The proportion of patients with ≥2 comorbidities did not differ significantly between the CCP and control groups (CCP: 49/92, 53.3%; No CCP: 74/132, 56.1%; Fisher’s exact test, *p* = 0.685). Due to heterogeneous and incomplete retrospective data, immunosuppression status could not be reliably characterized beyond the presence of malignancy (malignancy prevalence: 4.5% in controls vs 3.6% in the CCP group). Routine anti–SARS-CoV-2 antibody testing at admission was not performed (therefore, stratification by baseline serostatus was not possible).

Admission laboratory values (including CRP, D-dimers, complete blood count, creatinine, electrolytes, ALT/AST, and glucose) were comparable between groups (no significant differences). The median length of hospitalization was 13 days in the CCP group and 12 days in the control group.

During the study period, standard COVID-19 treatment primarily consisted of systemic glucocorticoids and, in a subset of patients, remdesivir. The proportions of patients receiving remdesivir and systemic glucocorticoids were higher in the CCP group (52.2% vs. 23.5% and 81.5% vs. 59.8%, respectively), whereas “supportive care only” (neither remdesivir nor glucocorticoids) was more common in the control group (31.1% vs. 6.5%). Tocilizumab and JAK inhibitors were not used in this cohort (they were not routinely used at the center during the study period). Information on other immunomodulators was not systematically recorded.

Individual-level data on SARS-CoV-2 variants were not available (sequencing was not routine). The recruitment period (October 2020 to January 2021) corresponds to earlier waves of the pandemic in Poland, which preceded the dominance of the Alpha variant; at that time, pre-Alpha lineages were predominant. Assigning a variant at the patient level was therefore not possible.

### 3.2. CCP Unit Characteristics and Transfusion Timing

Among 92 patients, a total of 117 units of CCP were administered (some patients received CCP in two separate transfusion episodes). Unit volume was typically ~250 mL (median 255 mL; IQR 250–261 mL in available unit records). Among 92 CCP recipients, 25 (27.2%) received two CCP units (two transfusion episodes). When two units were administered, they were typically issued/administered on the same day (median interval 0 days; range 0–1 day in records where both issue dates were available). Information on antibody titer/reactivity was available for 91/117 (77.8%) units; 26/117 (22.2%) had no titer data. Titer p/c (positive control ratio) refers to semi-quantitative anti-SARS-CoV-2 antibody reactivity reported by the transfusion service, obtained using Roche Elecsys electrochemiluminescence immunoassays (ECLIA). These assays measure binding antibody responses and are not equivalent to live-virus or pseudovirus neutralization assays; therefore, p/c values should be interpreted as serologic reactivity rather than functional neutralization. Among units with numerical values (*n* = 88), the median titer was 700 (IQR 400–1025; range 100–2000). The median time from admission to the first CCP transfusion was 6 days (IQR 4–8). The median time from collection to transfusion was 5 days (IQR 3–9).

### 3.3. Clinical Outcomes (Mortality, Intubation, Discharge)

Final outcome status was unavailable for 8 patients in the control group; therefore, crude death/discharge proportions were reported based on patients with complete information on hospital outcome.

Overall in-hospital mortality in the cohort was approximately 34%. Among patients with known outcomes, in-hospital mortality was 25% in the CCP group (23/92) versus 42% in controls (52/124; *p* = 0.010). The proportion of live discharges was higher in the CCP group (66% vs 50%; *p* = 0.022). Intubation and mechanical ventilation occurred in 17/224 (7.6%) patients, including 11/92 (12.0%) in the CCP group and 6/132 (4.5%) in the control group (*p* = 0.071). Because dates/times of intubation were not consistently recorded, time from admission to intubation could not be reliably determined.

In the basic logistic regression model adjusted for age and systemic glucocorticoid therapy, older age was independently associated with higher odds of in-hospital death (OR 1.04 per year; 95% CI 1.02–1.07; *p* < 0.001), whereas CCP administration remained associated with lower odds of death (OR 0.41; 95% CI 0.21–0.77; *p* = 0.005). Systemic glucocorticoid use showed a non-significant association with mortality in this model (OR 1.94; 95% CI 0.97–3.86; *p* = 0.061), likely reflecting confounding by indication (Table 2).

**Figure 1:**
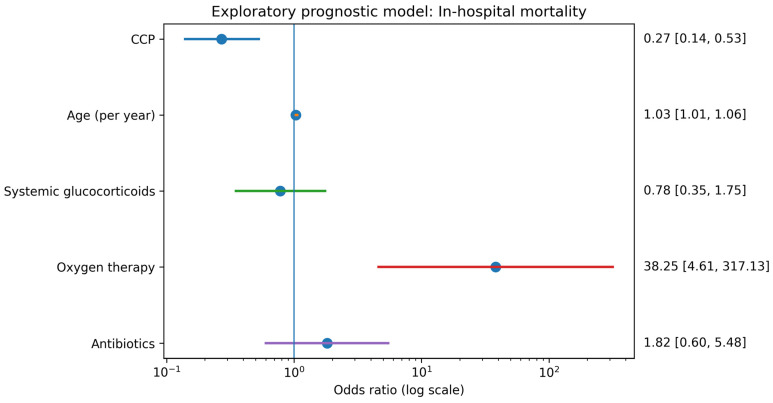
Forest plot for the exploratory prognostic model of in-hospital mortality including CCP, age, systemic glucocorticoids, oxygen therapy, and antibiotic therapy. Odds ratios with 95% confidence intervals are shown.

In the exploratory prognostic model additionally including oxygen therapy and antibiotic treatment during hospitalization as markers of disease severity and intensity of care, CCP remained independently associated with lower odds of in-hospital death (OR 0.27; 95% CI 0.14–0.53; *p* < 0.001) (Table 3, Figure 1). Age remained an independent predictor of mortality (OR 1.03 per year; 95% CI 1.01–1.06; *p* = 0.010). Oxygen therapy was strongly associated with higher odds of death (OR 38.25; 95% CI 4.61–317.13; *p* < 0.001), reflecting its role as a marker of severe respiratory failure. In this model, neither systemic glucocorticoid therapy (OR 0.78; 95% CI 0.35–1.75; *p* = 0.547) nor antibiotic use (OR 1.82; 95% CI 0.60–5.48; *p* = 0.289) was independently associated with mortality. Because oxygen therapy, antibiotics, and glucocorticoids could have been initiated in response to clinical deterioration, this model was interpreted as prognostic rather than causal.

In sensitivity analyses exploring alternative assumptions regarding the outcomes of the eight control patients with missing final status (all discharged alive, all deceased, or mortality proportion equal to that of controls with known outcomes), the relative risk estimates for in-hospital mortality associated with CCP remained consistent, ranging approximately from 0.55 to 0.63, with all corresponding *p* values < 0.05. 

In the basic logistic regression model adjusted for age, CCP administration, and remdesivir use, CCP was associated with higher odds of live hospital discharge (OR 2.03; 95% CI 1.08–3.82; *p* = 0.029). Older age was independently associated with lower odds of discharge (OR 0.96 per year; 95% CI 0.94–0.98; *p* < 0.001), whereas remdesivir use was not independently associated with discharge in this model (OR 1.17; 95% CI 0.60–2.26; *p* = 0.640) (Table 4).

**Figure 2:**
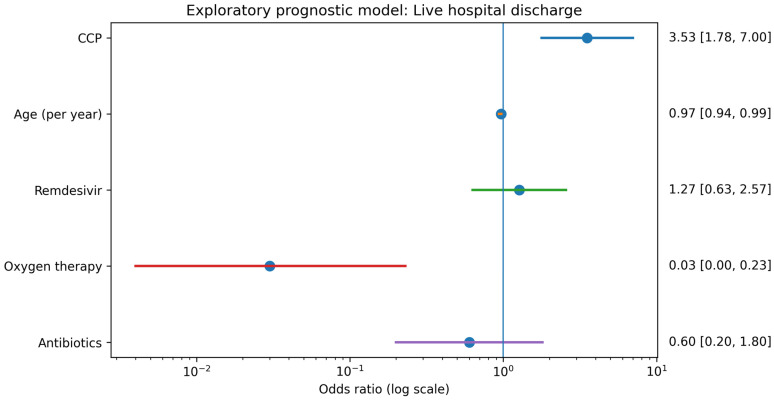
Forest plot for the exploratory prognostic model of live hospital discharge including CCP, age, remdesivir, antibiotic therapy, and oxygen therapy. Odds ratios with 95% confidence intervals are shown.

In the exploratory prognostic model additionally including oxygen therapy and antibiotic treatment during hospitalization as markers of disease severity and intensity of care, CCP remained strongly associated with higher odds of live discharge (OR 3.53; 95% CI 1.78–7.00; *p* < 0.001) (Table 5, Figure 2). Oxygen therapy was independently associated with substantially lower odds of discharge (OR 0.03; 95% CI 0.004–0.23; *p* < 0.001), reflecting its role as a marker of advanced respiratory failure. In this model, age, remdesivir use, and antibiotic therapy were not independently associated with discharge. As in the mortality analysis, variables dependent on the in-hospital course were interpreted prognostically rather than causally.

## 4. Discussion

In this retrospective, single-centre cohort from the early pandemic, transfusion of CCP was associated with a lower hazard of in-hospital death in time-dependent Cox models (HR 0.64; 95% CI 0.38–1.08) and with a higher probability of hospital discharge in multivariable analyses (OR 3.07; 95% CI 1.60–5.89). These associations remained robust in additional multivariable logistic regression models that explicitly adjusted for key in-hospital treatments, including systemic glucocorticoids in mortality models and remdesivir in discharge models. Age remained an independent predictor of mortality (OR per year 1.03; 95% CI 1.01–1.06). Directionally consistent results were observed across prespecified sensitivity approaches, including landmark analyses (days 3 and 5 after admission) and competing-risk analyses for discharge. Nevertheless, given the observational design, possible residual confounding (including confounding by indication) and the inherent risk of immortal-time bias—even with time-varying exposure modelling—our findings should be considered hypothesis-generating rather than definitive.

From a host–pathogen perspective, CCP is most biologically plausible as an antiviral intervention when administered within an early “viremic window”, before the hyper-inflammatory phase dominates clinical deterioration. The magnitude of its effect likely depends on (i) the potency of the product (SARS-CoV-2 antibody titre/neutralizing activity), (ii) timing of transfusion, and (iii) host factors such as baseline humoral immunity and immunosuppression.

Our observations should be interpreted in the context of randomized evidence. In the largest trial, RECOVERY, CCP did not reduce 28-day mortality in the overall population of hospitalized patients [15]. Differences between observational cohorts and RCTs may reflect heterogeneity in administration timing relative to symptom onset, reliance on variable antibody potency across CCP products, shifting standards of care across pandemic waves, and differences in baseline patient immunity. Importantly, updated systematic reviews and modern guidance increasingly converge on a narrow role of CCP: limited or no benefit in immunocompetent hospitalized populations, with potential usefulness according to external evidence and guidance mainly in selected patients with humoral immunodeficiency (a subset of the immunocompromised population)—particularly when high-titre product is administered early—and in selected outpatient settings where early treatment is feasible [16,17,18,19,20]. Because immunosuppression could not be quantified reliably in our cohort, this statement is provided for context and should not be interpreted as a subgroup-specific finding from our data.

Baseline clinical characteristics were broadly comparable between the CCP and control groups. In addition to similar prevalence of individual comorbidities, the overall burden of comorbidity—assessed as the proportion of patients with two or more comorbid conditions—did not differ significantly between groups. This reduces the likelihood that baseline multimorbidity confounded the observed associations between CCP administration and clinical outcomes. Systemic glucocorticoids were administered significantly more frequently in the CCP group. Because glucocorticoids have been shown to reduce mortality in hospitalized COVID-19 patients, their use was explicitly incorporated into both the basic and exploratory mortality models. After adjustment for glucocorticoid therapy, CCP administration remained independently associated with lower in-hospital mortality, suggesting that the observed association was not solely attributable to concomitant steroid treatment. In the exploratory prognostic model, glucocorticoid use itself was not independently associated with mortality after accounting for markers of disease severity, consistent with confounding by indication.

In our cohort, 117 CCP units were issued/transfused among 92 CCP-treated patients. Antibody titre/reactivity (“Titrep/c”) was documented for 91/117 units (77.8%), while 26/117 units (22.2%) lacked recorded unit-level titre data. A small number of results were recorded in a semi-quantitative format (e.g., “>800” and “>2000”), indicating heterogeneity in reporting. Among units with numeric titres available (*n* = 88), the median titre was 700 (IQR 400–1025; range 100–2000). While these data confirm that many administered units had measurable antibody reactivity, incomplete capture and non-uniform formats prevented robust dose–response analyses (e.g., high-titre vs low-titre comparisons) in relation to outcomes. Because antibody reactivity was assessed using immunoassays rather than functional neutralization tests, direct comparison with studies using live-virus neutralization titers should be made with caution.

Timing is another essential determinant of passive antibody efficacy. The median time from hospital admission to first CCP transfusion was 6 days (IQR 4–8). We were unable to define timing relative to symptom onset because symptom onset was not consistently recorded in retrospective documentation. As a result, admission was used as Time-0 and CCP was modelled as a time-varying exposure; landmark analyses at days 3 and 5 were additionally used to reduce immortal-time bias patterns typical for observational CCP studies. Baseline recipient anti-SARS-CoV-2 serology at admission was not routinely measured, and stored samples were not available for retrospective determination; therefore, we could not evaluate whether CCP preferentially benefited seronegative patients. In the absence of these variables, the level of respiratory support during hospitalization served as a pragmatic severity descriptor.

Remdesivir was also administered more frequently in the CCP group and may influence time to recovery. Accordingly, remdesivir use was incorporated into both the basic and exploratory models for live hospital discharge. Adjustment for remdesivir did not eliminate the association between CCP administration and a higher probability of discharge alive, indicating that this association was not driven solely by concomitant antiviral therapy.

Immunosuppression is a particularly important effect modifier in contemporary CCP evidence. In our dataset, malignancy was captured as a comorbidity; however, detailed information on active chemotherapy, systemic immunosuppressive therapy (e.g., biologics), transplant status, or B-cell-depleting regimens was not recorded in a sufficiently standardized manner to quantify the proportion of immunosuppressed patients reliably. This limitation restricts subgroup inference and complicates direct comparison with more recent data that specifically target immunocompromised populations.

Generalizability across pandemic waves is also constrained by viral evolution and population immunity. Our study reflects an early period (October 2020–January 2021), prior to Delta/Omicron and prior to widespread vaccination. Later in the pandemic, variant-dependent neutralization and the emergence of so-called Vax-CCP (plasma from vaccinated and/or previously infected donors) improved breadth against certain later subvariants [21]. Therefore, any extrapolation of these findings to subsequent waves should account for variant susceptibility, baseline population immunity, and the availability of alternative antivirals and monoclonal antibodies.

Regarding safety, CCP is administered as standard fresh frozen plasma and shares the known transfusion-related risks (e.g., TACO/TRALI), which are uncommon in contemporary reports. No new CCP-specific safety signals have been established in large observational safety cohorts, but appropriate patient selection and monitoring remain necessary [22,23].

Strengths and limitations. Strengths of this study include modelling CCP as a time-varying exposure, prespecified landmark analyses, competing-risk methods for discharge. Limitations include the single-centre retrospective design, moderate sample size, and incomplete availability/standardization of key variables needed to test biologically specific hypotheses: unit-level antibody potency for all CCP units, symptom onset timing, baseline recipient serology, and a structured immunosuppression profile. These constraints carry a practical “lessons learned” message for future emerging infectious diseases: early standardization of clinical documentation should include (1) intervention potency measures, (2) precise timing variables (symptom onset and intervention dates), (3) baseline host antibody status, and (4) clear, structured immunosuppression descriptors (including chemotherapy/biologic agents). Capturing these variables prospectively would substantially strengthen observational inference and accelerate platform RCT design and interpretation before large trial infrastructures are established.

In summary, in this early-pandemic cohort, CCP was associated with improved outcomes in time-dependent and multivariable models; however, causal inference is limited. In contemporary practice, based on modern evidence synthesis and professional guidance, the potential role of CCP is narrow and targeted—typically discussed in relation to patients with humoral immunodeficiency and early administration of a high-titer product—consistent with modern evidence synthesis and guidance. Importantly, our dataset did not capture immunosuppression in a sufficiently structured manner to quantify immunocompromised subgroups; therefore, these contemporary statements provide context rather than a subgroup-specific finding from this cohort.

Implications for practice/Regulatory context

Clinical implications: In the current landscape, the role of CCP—if any—should be narrow and targeted: early administration of an FDA-qualified/high-titer product in selected patients most likely to benefit (e.g., those with profound humoral immunodeficiency), particularly when alternative antivirals or variant-active monoclonal antibodies are unavailable or inactive against the prevailing variant. Because immunosuppression was not captured in a standardized way in our retrospective dataset, this practice-oriented statement reflects external evidence and guidance rather than a subgroup effect demonstrated in our cohort. For immunocompetent hospitalized patients, routine CCP is not recommended.

Regulatory context (USA, 2025): On 27 August 2025, the US FDA revoked the Emergency Use Authorization for CCP; the agency indicated it does not intend to object to the use of any remaining inventory distributed before revocation, whereas further use should proceed with appropriately authorized/licensed products in accordance with current regulatory requirements [24].

Clinical guidelines: According to IDSA, CCP is not recommended for routine use in hospitalized COVID-19 patients. A potential role is restricted to selected ambulatory, high-risk patients with mild-to-moderate disease when other effective options are unavailable, ideally within 8 days of symptom onset using FDA-qualified high-titer units (conditional recommendation, low certainty). Research needs include focusing on humoral immunodeficiency and incorporating baseline neutralizing-antibody testing.

## 5. Conclusions

In our single-center study with a retrospective cohort from the early phase of the pandemic, the use of CCP was associated with a lower risk of death (time-varying exposure Cox: HR 0.64; 95% CI 0.38–1.08) and higher odds of hospital discharge (multivariable model: OR 3.07; 95% CI 1.60–5.89), with these associations persisting after adjustment for key concomitant in-hospital therapies, including systemic glucocorticoids and remdesivir.

Age remained an independent, strong predictor of mortality (per-year OR 1.03; 95% CI 1.01–1.06).

Contemporary guidance suggests that the role of CCP—if any—is narrow and targeted (e.g., selected patients with significant humoral immunodeficiency, early administration, and high-titer products); our data provide a historical reference point for clinical practice during the first waves of the pandemic. However, immunocompromised status could not be quantified reliably in this retrospective cohort, limiting subgroup inference.

## Figures and Tables

**Table 1:** Patient characteristics and concomitant in-hospital treatments (CCP vs. control group). Continuous variables are presented as mean (SD) or median (IQR), and categorical variables as *n* (%). Between-group comparisons were performed using Student’s *t*-test or the Mann–Whitney U test, and the χ^2^ test or Fisher’s exact test.

Variable	No CCP (*n* = 132)	CCP (*n* = 92)	*p* Value
Age, mean (SD)	69.4 (14.8)	65.6 (15.1)	0.062
Hypertension (NT), %	52.0%	53.6%	0.314
Diabetes (DM), %	32.0%	22.3%	0.201
Obesity, %	29.7%	27.7%	0.556
COPD, %	12.0%	10.7%	0.945
Asthma, %	2.3%	1.8%	0.877
Chronic kidney disease, %	9.1%	9.8%	0.721
Cancer (neoplasm), %	4.5%	3.6%	0.811
Ischemic heart disease, %	9.8%	7.1%	0.523
Vascular disease, %	7.6%	6.2%	0.701
CRP (mg/L), median (IQR)	102.0 (44.0–171.0)	97.0 (40.0–160.0)	0.441
Hemoglobin (g/dL), median (IQR)	12.8 (11.6–14.0)	13.1 (11.8–14.4)	0.332
WBC (×10^3^/μL), median (IQR)	6.5 (4.7–9.4)	6.2 (4.6–8.7)	0.510
Lymphocytes (×10^3^/μL), median (IQR)	1.0 (0.6–1.5)	0.9 (0.6–1.4)	0.388
Platelets (×10^3^/μL), median (IQR)	215 (164–281)	225 (172–298)	0.472
Sodium (mmol/L), median (IQR)	138 (136–141)	139 (136–142)	0.398
Potassium (mmol/L), median (IQR)	4.2 (3.9–4.6)	4.1 (3.8–4.5)	0.421
D-dimers (ng/mL), median (IQR)	890 (490–1630)	850 (480–1580)	0.465
Creatinine (mg/dL), median (IQR)	0.98 (0.79–1.20)	0.95 (0.78–1.19)	0.372
ALT (U/L), median (IQR)	32 (21–47)	31 (20–45)	0.489
AST (U/L), median (IQR)	34 (25–47)	33 (24–45)	0.501
Glucose (mg/dL), median (IQR)	113 (98–132)	110 (96–128)	0.444
Hospital stay (days), median (IQR)	12 (8–18)	13 (9–19)	0.388
No oxygen/ventilatory support	33 (25.0%)	6 (6.5%)	<0.001
Oxygen therapy only	74 (56.1%)	51 (55.4%)	1.0
Non-invasive ventilation/HFNOT	19 (14.4%)	24 (26.1%)	0.0380
Invasive mechanical ventilation	6 (4.5%)	11 (12.0%)	0.0697
Time from hospital admission to CCP transfusion (days), median (IQR)	–	6 (4–8)	–
Remdesivir	31 (23.5%)	48 (52.2%)	<0.001
Systemic glucocorticoids (e.g., dexamethasone)	79 (59.8%)	75 (81.5%)	<0.001
Tocilizumab	NA	NA	–
No remdesivir and no systemic glucocorticoids (supportive care only)	41 (31.1%)	6 (6.5%)	<0.001

Abbreviations: CCP—COVID-19 convalescent plasma; NA—data not available. Baseline anti-SARS-CoV-2 antibody status at admission was not routinely measured and is therefore not available.

**Table 2:** Logistic regression for in-hospital mortality—basic model adjusted for age, CCP, and systemic glucocorticoids. Results are presented as ORs with 95% CIs.

Variable	OR	95% CI	*p* Value
CCP	0.41	0.21–0.77	0.005
Age (years)	1.04	1.02–1.07	<0.001
Systemic glucocorticoids	1.94	0.97–3.86	0.061

**Table 3:** Logistic regression for in-hospital mortality—exploratory prognostic model including in-hospital severity and treatment markers (CCP, age, systemic glucocorticoids, antibiotic therapy, and oxygen therapy). Results are presented as ORs with 95% CIs. Variables dependent on the in-hospital course were interpreted prognostically.

Variable	OR	95% CI	*p* Value
CCP	0.27	0.14–0.53	<0.001
Age (years)	1.03	1.01–1.06	0.010
Systemic glucocorticoids	0.78	0.35–1.75	0.547
Antibiotics	1.82	0.60–5.48	0.289
Oxygen therapy	38.25	4.61–317.13	<0.001

**Table 4:** Logistic regression for live hospital discharge—basic model adjusted for age, CCP, and remdesivir. Results are presented as odds ratios (ORs) with 95% confidence intervals (CIs).

Variable	OR	95% CI	*p* Value
CCP	2.03	1.08–3.82	0.029
Age (years)	0.96	0.94–0.98	<0.001
Remdesivir	1.17	0.60–2.26	0.640

**Table 5:** Logistic regression for live hospital discharge—exploratory prognostic model including in-hospital severity and treatment markers (CCP, age, remdesivir, antibiotic therapy, and oxygen therapy). Results are presented as ORs with 95% CIs. Variables dependent on the in-hospital course were interpreted prognostically.

Variable	OR	95% CI	*p* Value
CCP	3.53	1.78–7.00	<0.001
Age (years)	0.97	0.94–0.99	0.012
Remdesivir	1.27	0.63–2.57	0.502
Antibiotics	0.60	0.20–1.80	0.361
Oxygen therapy	0.03	0.004–0.23	<0.001

## Data Availability

Detailed information will be provided upon request via email at: k.kalinowska@umw.edu.pl.
